# “THEY DON’T TELL YOU ANYTHING”: ETHICS OF PRIVACY AND SOCIAL RELATIONSHIPS IN ASSISTED LIVING

**DOI:** 10.1093/geroni/igz038.3327

**Published:** 2019-11-08

**Authors:** Emma Cooke, Molly M Perkins, Patrick Doyle, Kathy Kinlaw, Kevin Wack, Ann E Vandenberg

**Affiliations:** 1 Division of General Medicine and Geriatrics, Emory University School of Medicine, Atlanta, Georgia, United States; 2 Center for Innovative Care in Aging, Johns Hopkins School of Nursing, Baltimore, Maryland, United States; 3 Emory University Center for Ethics, Atlanta, Georgia, United States

## Abstract

The Health Insurance Portability and Accountability Act was developed to ensure patient privacy. Yet in assisted living (AL), social connectedness—which is associated with positive sense of self and well-being—may conflict with privacy regulations restricting information sharing. These regulations, while intended to protect, rely on a traditional conception of autonomy that foregrounds self-determination, freedom of choice, and freedom from outside interference, rather than a relational definition that acknowledges dependency, interdependence, and care relationships. We sought to identify health information sharing practices in AL that help or hinder residents’ ability to maintain a positive sense of self. We conducted a thematic analysis with secondary data (61 interviews with residents and their informal and formal caregivers, 916 hours of ethnographic observation) from one large (125 beds) AL community in Atlanta enrolled in a 5-year NIA-funded end-of-life study (5R01AG047408). We examined these data to determine how health information is shared in AL, and the valence of different sharing practices. Findings showed that exchanging information about shared life stage and health circumstances built community within AL. Conversely, receiving partial or inadequate health information frustrated residents. Medical information could be inferred from environmental cues, but many residents felt these cues harmfully “medicalized” social space. Negotiating privacy boundaries required staff and resident compromise. These results indicate divergence between AL policies intended to preserve privacy, and resident values emphasizing social relationships and bonding. A relational perspective may be valuable in developing alternatives for residents wishing to share health information with peers.

